# Contribution of 3H-thymidine labelling index and flow cytometric S-phase in predicting survival of patients with non-Hodgkin's lymphoma.

**DOI:** 10.1038/bjc.1992.337

**Published:** 1992-10

**Authors:** A. Costa, R. Silvestrini, R. Giardini, G. Messina-Gabrielli, P. Boracchi, S. Veneroni

**Affiliations:** Oncologia Sperimentale C, Istituto Nazionale per lo Studio e la Cura dei Tumori, Milan, Italy.

## Abstract

The 3H-thymidine labelling index (3H-dT LI) of cell suspensions from fresh material and the flow cytometric S-phase (FCM-S) of nuclei recovered from paraffin blocks were determined on the same pathologic lymph node specimen for 190 non-Hodgkin's lymphomas (NHLs). FCM-S was defined by a planimetric method and by an optimization procedure. Poor correlation coefficients were observed among the three cell kinetic variables. All three cell kinetic variables were significant indicators of 8-year survival and median survival time. The life-regression procedure evidenced a significant relative contribution of 3H-dT LI and FCM-S, thus suggesting a different biologic meaning of the two cell kinetic variables. This finding was further supported by evidence that simultaneous use of 3H-dT LI and FCM-S can identify groups of patients with different survival better than when either modality is used alone. Multivariate analysis indicated that the risk groups as defined by cell kinetic variables are predictors of survival even in the presence of established factors such as histology and stage.


*Br. J. Cancer* (1992), **66**, 680–684 © Macmillan Press Ltd., 1992

### Contribution of $^{3}$H-thymidine labelling index and flow cytometric S-phase in predicting survival of patients with non-Hodgkin's lymphoma

A. Costa[1], R. Silvestrini[1], R. Giardini[2], G. Messina-Gabrielli[1], P. Boracchi[3] & S. Veneroni[1]

[1]*Oncologia Sperimentale C,* [2]*Anatomia Patologica, and* [3]*Biometria, Istituto Nazionale per lo Studio e la Cura dei Tumori, Milan, Italy.*

**Summary** The $^{3}$H-thymidine labelling index ($^{3}$H-dT LI) of cell suspensions from fresh material and the flow cytometric S-phase (FCM-S) of nuclei recovered from paraffin blocks were determined on the same pathologic lymph node specimen for 190 non-Hodgkin's lymphomas (NHLs). FCM-S was defined by a planimetric method and by an optimization procedure. Poor correlation coefficients were observed among the three cell kinetic variables. All three cell kinetic variables were significant indicators of 8-year survival and median survival time. The life-regression procedure evidenced a significant relative contribution of $^{3}$H-dT LI and FCM-S, thus suggesting a different biologic meaning of the two cell kinetic variables. This finding was further supported by evidence that simultaneous use of $^{3}$H-dT LI and FCM-S can identify groups of patients with different survival better than when either modality is used alone. Multivariate analysis indicated that the risk groups as defined by cell kinetic variables are predictors of survival even in the presence of established factors such as histology and stage.

The prognostic relevance of cell kinetics in malignant non-Hodgkin's lymphomas (NHLs) has been the subject of study for several decades. Cell kinetic studies have consistently shown that $^{3}$H-thymidine labelling index ($^{3}$H-dT LI) is an indicator of long-term clinical outcome (Brandt *et al.*, 1981; Costa *et al.*, 1981; Kvaloj *et al.*, 1985), independently of prognostic factors such as histopathology and stage (Silvestrini *et al.*, 1989).

More recently, we showed that the use of bromodeoxyuridine (BUdR) on NHL specimens gives labelling index values superimposable to those obtained with $^{3}$H-thymidine (Silvestrini *et al.*, 1988). However, both approaches are based on the incorporation of DNA metabolic precursors and therefore imply the availability of fresh and viable tumour material. Such a requirement cannot always be satisfied when, as often happens, patients are referred with pathologic material already fixed and another biopsy is not ethically justified or easily obtainable. Moreover, determination of $^{3}$H-dT LI or BUdR LI is limited to prospective collection of fresh biopsies and precludes retrospective analysis of paraffin-embedded samples from series of patients with a long follow-up.

Several studies have shown that the flow cytometric S-phase cell fraction (FCM-S) is a discriminant of clinical outcome in NHL patients (Braylan *et al.*, 1980; Scarffe & Crowther, 1981; Roos *et al.*, 1985; Christensson *et al.*, 1986; Lenner *et al.*, 1987; Griffin *et al.*, 1988; Grierson *et al.*, 1990; Rehn *et al.*, 1990; Joensuu *et al.*, 1990). Flow cytometric analysis is an automatic and quick method to measure the S-phase cell fraction, and an advantage is that stocked paraffin blocks can be used for retrospective analysis.

The present study compared the results obtained when the S-phase cell fraction was measured by autoradiography and by flow cytometry with two mathematical models, i.e., a planimetric method and an optimisation procedure. Moreover, through univariate and multivariate analyses we defined the prognostic power for survival of cell kinetics and clinical and pathologic variables determined in parallel on the same patients.

Correspondence: R. Silvestrini, Oncologia Sperimentale C, Istituto Nazionale Tumori, Via Venezian 1, 20133 Milan, Italy.
Presented in part at the Annual Meeting of the Cell Kinetics Society, March 21–24, 1991. Charleston, SC.
Received 22 November 1991; and in revised form 20 March 1992.

### Materials and methods

### Case series

Biopsies were obtained from 190 patients with NHLs at the National Cancer Institute of Milan between 1974 and 1989. The $^{3}$H-dT LI was determined on fresh tissue specimens, and flow cytometric S-phase was determined on the same biopsy sample from paraffin-embedded blocks. All patients were pathologically staged and classified according to the Ann Arbor Staging System (Carbone *et al.*, 1971). Treatment was decided according to different prospective protocols (Monfardini *et al.*, 1980; Monfardini *et al.*, 1984; Bajetta *et al.*, 1988; Tondini *et al.*, 1991) or to the best standard therapy.

Histologic specimens were retrospectively reviewed and classified according to the Working Formulation for clinical usage according with The Non-Hodgkin's Lymphoma Pathologic Classification Project (1982). The new National Cancer Institute clinical distinction (NCI system) between indolent lymphoma (small lymphocytic; follicular, small cleaved cells; follicular, mixed; diffuse, small cleaved cells) and aggressive lymphoma (follicular, large cell; diffuse, mixed; diffuse, large cell; diffuse, immunoblastic) was applied for prognostic analysis (De Vita *et al.*, 1989; Urba *et al.*, 1990).

### $^{3}$H-dT LI

$^{3}$H-dT LI was determined on cell suspensions obtained by mechanical disaggregation of lymph node material as previously described (Costa *et al.*, 1981). Briefly, $6 \times 10^{6}$ cells were incubated with $^{3}$H-thymidine (4 $\mu$Ci; specific activity, 25 Ci mmol$^{-1}$; Radiochemical Centre, Amersham, UK) for 1 h at 37°C. Autoradiography was performed according to the stripping film (AR10 Kodak) technique. The $^{3}$H-dT LI was determined on the entire cell population by scoring 5,000–10,000 cells from different cytospins for each tumour.

### FCM-S

Flow cytometric analysis was performed on cells recovered from paraffin-embedded specimens and fixed in formalin (132 cases) or Zenker's solution (58 cases). Tumor sample adequacy was assessed at the microscope on hematoxylin-eosin-stained sections adjacent to those used for flow cytometric analysis and was verified by a pathologist (R.G.).

The technique of Hedley *et al.* (1983) with minor modifications was used. The nuclei suspension was stained with a solution containing propidium iodide (Calbiochem, Boehring, Corp., San Diego, CA) at the concentration of 50 µm $ml^{-1}$ in $H_2O$, 0.05% Nonidet P40 (Calbiochem) and RNase (Type IA, from bovine pancreas, Sigma).

DNA analysis was performed using a FACScan flow cytometer (Becton-Dickinson, San Jose, CA), and a minimum of 30,000 events was acquired on a single-parameter 256-channel integrated fluorescence histogram. Since the use of paraffin-embedded specimens does not allow the use of an internal standard to determine the position of a diploid $G_{0/1}$ population of cells (Schute *et al.*, 1985), an aneuploid cell population was considered to be present only if two distinct $G_{0/1}$ peaks were evident. In such DNA profiles, the DNA index of the aneuploid population was calculated by dividing the mean fluorescence channel of the $G_{0/1}$ peak with the higher intensity by the mean fluorescence channel of the $G_{0/1}$ peak with the lower intensity. A lower median CV was observed for formalin-fixed (4.9%) than for Zenker-fixed specimens (8.9%). Only DNA profiles with a $G_{0/1}$ peak CV less than 8% were considered evaluable, and this criterion was fulfilled in 155 of the original 190 cases.

FCM-S was computed by two mathematical models: a planimetric method (RFIT, Becton Dickinson) and an optimisation procedure (Lampariello & Del Bino, 1988). According to the first model the S phase was calculated from a rectangle, where the height of the rectangle is the average height of mid S phase. For aneuploid tumors, the S-phase fraction of the aneuploid population alone was calculated [i.e. (number of aneuploid S-phase cells/total number of aneuploid cells) × 100]. The second model represents an optimisation procedure to process DNA histograms containing up to two cell populations and includes an exponential function to subtract background debris. An objective measure of goodness of fit is obtained by computing the chi-square error index. In our experience, chi-square values lower than 3 corresponded to nearly exact fits, as judged by visual inspection.

*Statistical analysis*

Spearman's correlation coefficient (Hajek & Sidak, 1967) was used to compare S-phase percentages obtained by the two models and to investigate the relationship between FCM-S and $^3$H-dT LI. The differences in cell kinetics in the various clinico-pathologic subsets were analysed by means of the Wilcoxon rank-sum test.

The role of each of the prognostic variables (univariate analysis) and their joint effect (multivariate analysis) were evaluated by resorting to a Weibull regression model. In this model each regression coefficient (β) is recognisable as the log of the hazard ratio and it is constant with time. For each variable, the 'unadjusted' hazard ratio and its 95% confidence interval were estimated using the putative 'best prognosis' as reference category. To investigate the joint prognostic relevance of the kinetic variables ($^3$H-dT LI, planimetric and optimisation FCM-S), the 'adjusted' hazard ratios were estimated by resorting to a multiple regression model containing the three variables. The parsimonious regression model was obtained by means of a backward elimination procedure.

### Results

FCM-S was computed by the planimetric method and the optimisation procedure on 149 of 155 cases with evaluable DNA histograms. Similar median values (3.7% and 4.0%), ranges and exponential distributions were observed for $^3$H-dT LI and FCM-S determined by the optimisation procedure, which implies subtraction of debris contamination. A higher median FCM-S value (12%), as expected, and a log-normal distribution were observed by using the planimetric method without background subtraction.

Statistically significant correlation coefficients between the $^3$H-dT LI values and the FCM-S values obtained by the two

**Figure 1** Relationship between cell kinetic variables. **a**, $^3$H-dT LI *vs* planimetric FCM-S; **b**, $^3$H-dT LI *vs* optimisation FCM-S; **c**, planimetric *vs* optimisation FCM-S. $r_s$ = Spearman's correlation coefficient.

quantification approaches were observed. However, the strength of the association appeared to be rather poor (Figure 1a,b). A significant correlation with a somewhat higher correlation coefficient ($r_s = 0.70$) was also observed between the FCM-S data pairs obtained with or without background subtraction (Figure 1c). Cell kinetics, expressed in the three different ways, was consistently related to histology as defined by the NCI system and unrelated to stage or patient's age. Conversely, FCM S-phase fraction, but not $^3$H-dT LI, was related to DNA ploidy (Table I).

Survival analysis was performed on the 138 patients with complete kinetic evaluation and follow-up information. The median value of each cell kinetic variable was used as the cutoff between slowly and rapidly proliferating NHLs. Univariate analysis showed that all three variables were correlated to 8-year survival but with a different discriminant power. In fact, survival curves were more diversified for the two kinetic subgroups as defined by $^3$H-dT LI than for those defined by FCM-S (Figure 2). Patients with high $^3$H-dT LI tumours had about a four-fold shorter median survival than those with low $^3$H-dT LI tumours (Table II). Conversely, the difference was only about two-fold when the FCM-S determination was considered. These findings were also reflected

**Table I** Relationship between cell kinetic variables and biologic, clinico-pathologic features

| | $^3$H-dT LI (%) | | FCM-S (median, %) | | | |
|---|---|---|---|---|---|---|
| | *Median* | *P value* | *Optimisation procedure* | *P value* | *Planimetric method* | *P value* |
| *Working Formulation*[a] | | | | | | |
| Indolent lymphoma | 2.0 | <0.001 | 3.0 | <0.001 | 10.0 | <0.001 |
| Agressive lymphoma | 9.6 | | 10.3 | | 20.3 | |
| *Stage* | | | | | | |
| I–II | 4.5 | ns | 3.8 | ns | 12.1 | ns |
| III–IV | 3.5 | | 4.5 | | 12.2 | |
| *Patient's age* | | | | | | |
| ⩽60 yrs | 3.6 | ns | 4.4 | ns | 11.1 | ns |
| >60 yrs | 5.1 | | 4.3 | | 13.0 | |
| *DNA ploidy* | | | | | | |
| Diploid | 4.6 | ns | 3.8 | 0.05 | 10.8 | <0.001 |
| Aneuploid | 3.5 | | 5.8 | | 20.0 | |

[a]According to NCI system.

**Table II** Prognostic relevance for survival of cell kinetic variables

| | *Median survival (mo)* | *Hazard ratio* | *95% Confidence interval* | *P*[b] *value* |
|---|---|---|---|---|
| *$^3$H-dT LI* | | | | |
| ⩽4%[a] | 80 | 2.38 | 1.67–3.39 | 0.001 |
| >4% | 17 | | | |
| *FCM-S* | | | | |
| Planimetric method | | | | |
| ⩽12%[a] | 62 | 1.96 | 1.21–3.16 | 0.006 |
| >12% | 25 | | | |
| Optimisation procedure | | | | |
| ⩽4%[a] | 59 | 1.62 | 0.98–2.68 | 0.05 |
| >4% | 26 | | | |

[a]Reference category. [b]Based on Wald statistics.

by the different hazard ratios observed when considering the three different variables (Table II). Relative influence on survival of the three cell kinetic variables was assessed by a multiple regression analysis (Table III). Using a backward method for regression, the value of FCM-S according to the optimisation procedure was removed from the model, leaving $^3$H-dT LI and planimetric FCM-S in the final step. These two cell kinetic variables retained independent prognostic values even though they were characterised by a different hazard ratio and effect on long-term survival (Table III). Finally, we evaluated whether information provided by these two cell kinetic variables could identify groups of patients with different survival probabilities better than either modality alone. Plots of the survival curve obtained by the regression model identified three groups of patients with different survival probabilities. Survival curves estimated by the regression model for each group showed that patients with two unfavourable cell kinetic variables (high $^3$H-dT LI, high FCM-S) did very poorly, with a median survival of only 12 months (Figure 3). In contrast, the median survival of patients with favourable variables (low $^3$H-dT LI, low FCM-S) was 80 months, and the presence of only one risk factor identified a group with an intermediate median survival of 59 months. Multiple regression analysis of cell kinetic variables, indolent and aggressive lymphomas as defined by the NCI system, and Ann Arbor stage (Table IV) showed that in the present series of patients cell proliferation index retained its prognostic relevance.

### Discussion

The proliferation indices, as defined by flow cytometric and autoradiographic methods, have shown their relevance as prognostic indicators in NHLs (Braylan *et al.*, 1980; Brandt *et al.*, 1981; Costa *et al.*, 1981; Scarffe & Crowther 1981; Kvaloj *et al.*, 1985; Roos *et al.*, 1985; Christensson *et al.*, 1986; Lenner *et al.*, 1987; Griffin *et al.*, 1988; Silvestrini *et al.*, 1989; Grierson *et al.*, 1990; Rehn *et al.*, 1990; Joensuu *et al.*, 1990) as well as in other human malignancies (Volm *et al.*, 1985; Volm *et al.*, 1988; Costa *et al.*, 1990; McGuire *et al.*, 1990; O'Reilly *et al.*, 1990; Silvestrini *et al.*, 1990). However, cell kinetic variables have been usually studied independently on different series of patients, thus preventing a clear comparison between the different methods. The present study allowed a direct comparison, on the same biopsy sample, of

**Figure 2** Eight-year survival as a function of cell kinetic variables. **a**, $^3$H-dT LI; **b**, planimetric FCM-S; **c**, optimisation FCM-S. (----) slowly proliferating NHLs; (——) rapidly proliferating NHLs.

**Table III** Multiple regression analysis of cell kinetic variables

| Model | Chi square[a] | P value | Hazard ratio | 95% confidence interval |
|---|---|---|---|---|
| *Complete* | | | | |
| $^3$H-dT LI (>4% *vs* ≤4%) | 5.50 | 0.02 | 1.83 | 1.11–3.04 |
| *FCM-S* | | | | |
| Planimetric method (>12% *vs* ≤12%) | 3.26 | 0.07 | 1.65 | 0.96–2.85 |
| Optimisation procedure (>4% *vs* ≤4%) | 0.23 | n.s. | 1.14 | 0.66–1.97 |
| *Final* | | | | |
| $^3$H-dT LI | 8.04 | 0.005 | 1.96 | 1.23–3.13 |
| Planimetric FCM-S | 3.96 | 0.046 | 1.63 | 1.01–2.63 |

[a]Based on Wald statistics.

**Figure 3** Eight-year survival as a function of combined $^3$H-dT LI and FCM-S. Low proliferation index (43 patients): $^3$H-dT LI ≤4% and FCM-S ≤12%; intermediate proliferation index (46 patients): $^3$H-dT LI ≤4% and FCM-S >12%, or $^3$H-dT LI >4% and FCM-S ≤12%; high proliferation index (50 patients): $^3$H-dT LI >4% and FCM-S >12%.

the interrelationship and of the relative contribution as prognostic indicators of $^3$H-dT LI and of FCM-S.

The study showed the high feasibility, which was favoured by the high frequency of diploid NHLs (about 70%), of FCM-S determination for paraffin-embedded specimens. Moreover, in agreement with the results of several studies (Griffin *et al.*, 1988; Grierson *et al.*, 1990; Joensuu *et al.*, 1990), the clinical relevance of ploidy was not found (data not shown) and the relevance of FCM-S as a prognostic indicator for survival was confirmed. The results also showed a greater reliability of a planimetric method than of a more sophisticated optimisation procedure with background subtraction to quantify the S-phase cell fraction. We interpret these findings as evidence that exponential debris subtraction could be of greater biologic relevance in other tumor systems than in NHLs. Although debris and contaminating non-neoplastic populations both interfere with FCM-S quantifications, the relative artifactual contribution of each alteration of a true neoplastic proliferative cell fraction is not known. The effect of both variables most likely varies considerably from case to case, depending on a number of factors intrinsic to the individual tumour and technical in nature.

**Table IV** Multiple regression analysis for survival

| | Chi square[a] | P value |
|---|---|---|
| $^3$H-dT LI, FCM-S | 7.12 | <0.05 |
| Stage | 2.31 | ns |
| Working Formulation[b] | 1.04 | ns |

[a]Based on Wald statistics. [b]According to NCI system.

Furthermore, the present study suggests that the different accuracy of flow cytometric or autoradiographic methods in predicting clinical outcome is probably due to a different biologic meaning of the two cell kinetic variables. This assumption is supported by their difference in identifying proliferating cells. In fact, $^3$H-dT as a precursor of thymine is therefore a specific precursor of DNA and it has a functional activity, i.e., it measures the fraction of cycling S-phase cells. As regards FCM-S, such a determination does not discriminate between actually DNA synthesising cells and those with an S-phase equivalent DNA content but quiescent. Moreover, the relation observed between DNA ploidy and the S-phase cell fraction defined on the basis of nuclear DNA content and the lack of a relation with the S-phase cell fraction defined by DNA precursor incorporation could indicate that the two cell kinetic variables are partially complementary. In fact, in combination they accurately identify subgroups at different risk.

Multiple regression analysis including cell kinetic variables, histology (as defined by the NCI system or by the three subsets of the Working Formulation), and stage indicated the relevance of the association of $^3$H-dT LI and FCM-S in predicting survival. However, owing to the heterogeneity of our population of NHL patients and the different therapeutic approaches used during the study period, we do not feel that the present study can define the correlation between kinetic variables and complete remission. Therefore, the precise role of kinetic variables in defining survival should be interpreted with caution. These findings should be confirmed by prospective studies on adequate series of patients homogeneous for tumour histology and treatment. Such an analysis should necessarily include other biologic factors of established prognostic relevance, such as LDH and $\beta_2$-microglobulin, number of extranodal sites and presence of bulky disease.

The authors are grateful to Ms G. Abolafio for excellent technical assistance and to Ms P. Rinaldi for secretarial assistance.

### References

BRANDT, L., OLSSON, H. & MONTI, M. (1981). Uptake of thymidine in lymphoma cells obtained through fine needle aspiration biopsy. Relation to prognosis in non-Hodgkin's lymphomas. *Eur. J. Cancer Clin. Oncol.*, **17,** 1229–1233.

BRAYLAN, R.C., DIAMOND, L.W., POWELL, M.L. & HARTY-GOLDER, B. (1980). Percentage of cells in the S-phase of the cell cycle in human lymphomas determined by flow cytometry: correlation with labeling index and survival. *Cytometry*, **1,** 171–174.

BAJETTA, E., VALAGUSSA, P. & BONADONNA, G. (1988). Combined modality treatment for stage I-II non-Hodgkin's lymphomas: CVP versus BACOP chemotherapy. *Int. J. Radiat. Oncol. Biol. Phys.*, **15**, 3-12.

CARBONE, P.P., KAPLAN, H.S., MUSSHOFF, K., SMITHERS, D.W. & TUBIANA, M. (1971). Report of the Committee on Hodgkin's Disease Staging Classification. *Cancer Res.*, **31**, 1860-1861.

CHRISTENSSON, B., TRIBUKAIT, B., LINDER, I.L., ULLMAN, B. & BIBERFELD, P. (1986). Cell proliferation and DNA content in non-Hodgkin's lymphoma. *Cancer*, **58**, 1295-1304.

COSTA, A., BONADONNA, G., VILLA, E., VALAGUSSA, P. & SILVESTRINI, R. (1981). Labeling index as a prognostic marker in non-Hodgkin's lymphomas. *J. Natl Cancer Inst.*, **66**, 1-5.

COSTA, A., SILVESTRINI, R., MEZZANOTTE, G., VAGLINI, M., GRIGNOLIO, E., CLEMENTE, C. & CASCINELLI, N. (1990). Cell kinetics: an independent prognostic variable in stage II melanoma of the skin. *Br. J. Cancer*, **62**, 826-829.

DE VITA, V.T., JAFFE, E.S., MAUCH, P. & LONGO, D.L. (1989). Lymphocytic lymphoma. In *Cancer: Principles and Practice of Oncology*. De Vita, Hellman & Rosenberg (eds) pp. 1741-1798. Lippincott: Philadelphia.

GRIERSON, H.L., WOOLDRIDGE, T.N., PURTILO, D.T., PIERSON, J., BAST, M., WOOLDRIDGE, L., ARMITAGE, J.O. & WEISENBURGER, D.D. (1990). Low proliferative activity is associated with a favourable prognosis in peripheral T-cell lymphoma. *Cancer Res.*, **50**, 4845-4848.

GRIFFIN, N.R., HOWARD, M.R., QUIRKE, P., BRIEN, C.J.O. & CHILD, J.A. (1988). Prognostic indicators in centroblastic-centrocytic lymphoma. *J. Clin. Pathol.*, **41**, 866-870.

HAJEK, J. & SIDAK, Z. (1967). *Theory of the Rank Tests*. Academic Press: New York.

HEDLEY, D.W., FRIEDLANDER, M.L., TAYLOR, I.W., RUGG, C.A. & MUSGROVE, E.A. (1983). Method for analysis of cellular DNA content of paraffin-embedded pathological material using flow cytometry. *J. Histochem. Cytochem.*, **31**, 1333-1335.

JOENSUU, H., KLEMI, P.J. & JALKANEN, S. (1990). Biologic progression in non-Hodgkin's lymphoma. A flow cytometric study. *Cancer*, **65**, 2564-2571.

KVALOJ, S., MARTON, P., KAALHUS, O., HIE, J., FOSS-ABRAHAMSEN, A. & GODAL, T. (1985). $^3$H-Thymidine uptake in B cell lymphomas. Relation to treatment response and survival. *Scand. J. Haematol.*, **34**, 429-435.

LAMPARIELLO, F. & DEL BINO, G. (1988). Automatic parameter estimation of flow cytometric DNA distributions in the study of tumor cell kinetics. In *Identification and System Parameter Estimation*. Chen, H.F. (ed) pp. 1767-1772. Pergamon Press: New York.

LENNER, P., ROOS, G., JOHANSSON, H., LINDH, J. & DIGE, U. (1987). Non-Hodgkin lymphoma. Multivariate analysis of prognostic factors including fraction of S-phase cells. *Acta Oncol.*, **26**, 179-183.

MCGUIRE, W.L., TANDOM, K., CRAIG ALLRED, D., CHAMNESS, G.C. & CLARK, G.M. (1990). How to use prognostic factors in axillary node negative breast cancer patients. *J. Natl Cancer Inst.*, **82**, 1006-1015.

MONFARDINI, S., BANFI, A., BONADONNA, G., RILKE, F., MILANI, F., VALAGUSSA, P. & LATTUADA, A. (1980). Improved five-year survival after combined radiotherapy-chemotherapy for stage I-II non-Hodgkin's lymphoma. *Int. J. Radiation Oncol. Biol. Phys.*, **6**, 125-134.

MONFARDINI, S., RILKE, F., VALAGUSSA, P., BAJETTA, E., CANETTA, R., BUZZONI, R., GIARDINI, R. & VIVIANI, S. (1984). A clinicopathologic study in advanced non-Hodgkin's lymphomas treated with sequential non-cross-resistant regimens: comparison of the Working Formulation with the Rappaport and Kiel classifications. *Eur. J. Cancer Clin. Oncol.*, **5**, 609-617.

O'REILLY, S.M., CAMPLEJOHN, R.S., BARNES, D.M., MILLIS, R.R., ALLEN, D., RUBENS, R.D. & RICHARDS, M.A. (1990). DNA index, S-phase fraction, histological grade and prognosis in breast cancer. *Br. J. Cancer*, **61**, 671-674.

REHN, S., GLIMELIUS, B., STRANG, P., SUNDSTROM, C. & TRIBUKAIT, B. (1990). Prognostic significance of flow cytometry studies in B-cell non-Hodgkin's lymphoma. *Hematol. Oncol.*, **8**, 1-12.

ROOS, G., DIGE, U., LENNER, P., LINDH, J. & JOHANSSON, H. (1985). Prognostic significance of DNA-analysis by flow cytometry in non-Hodgkin's lymphoma. *Hematol. Oncol.*, **3**, 233-242.

SCARFFE, J.H. & CROWTHER, D. (1981). The pre-treatment proliferative activity of non-Hodgkin's lymphoma cells. *Eur. J. Cancer*, **17**, 99-108.

SCHUTE, B., REYNDERS, M.M.J., BOSMAN, F.T. & BLIJHAM, G.M. (1985). Flow cytometric determination of DNA ploidy level in nuclei isolated from paraffin-embedded tissue. *Cytometry*, **6**, 26-30.

SILVESTRINI, R., COSTA, A., VENERONI, S., DEL BINO, G. & PERSICI, P. (1988). Comparative analysis of different approaches to investigate cell kinetics. *Cell Tissue Kinet.*, **21**, 123-131.

SILVESTRINI, R., COSTA, A., GIARDINI, R., BORACCHI, P., DEL BINO, G., MARUBINI, E. & RILKE, F. (1989). Prognostic implications of cell kinetics, histopathology and pathologic stage in non-Hodgkin's lymphomas. *Hematol. Oncol.*, **7**, 411-422.

SILVESTRINI, R., DAIDONE, M.G., VALAGUSSA, P., DI FRONZO, G., MEZZANOTTE, G., MARIANI, L. & BONADONNA, G. (1990). $^3$H-Thymidine labeling index as a predictor in node-positive breast cancer. *J. Clin. Oncol.*, **8**, 1321-1326.

THE NON-HODGKIN'S LYMPHOMA PATHOLOGIC CLASSIFICATION PROJECT (1982). National Cancer Institute sponsored study of classifications of non-Hodgkin's lymphomas. Summary and description of a Working Formulation for clinical usage. *Cancer*, **49**, 2112-2135.

TONDINI, C., BUZZONI, R., VALAGUSSA, P., ROCCA, A., BENGALA, C., BANFI, A., ZANINI, M., LOMBARDI, F. & GIARDINI, R. (1991). Short-term chemotherapy (CHOP) followed by local-regional RT for localized non-Hodgkin's lymphoma. *Proc. ASCO*, **10**, 983.

URBA, W.J., DUFFEY, P.L. & LONGO, D.L. (1990). Treatment of patients with aggressive lymphomas: an overview. *J. Natl Cancer Inst. Monogr.*, **10**, 29-37.

VOLM, M., BRUGGERMANN, A., GUNTHER, M., KLEINE, W., PFEIDERER, A. & VOGH-SCHADEN, M. (1985). Prognostic relevance of ploidy, proliferation and resistance-predictive tests in ovarian carcinoma. *Cancer Res.*, **45**, 5180-5185.

VOLM, M., MATTERN, J., MULLER, T. & DRINGS, P. (1988). Flow cytometry of epidermoid lung carcinomas: relationship of ploidy and cell cycle phases to survival. A five-year follow-up study. *Anticancer Res.*, **8**, 105-112.


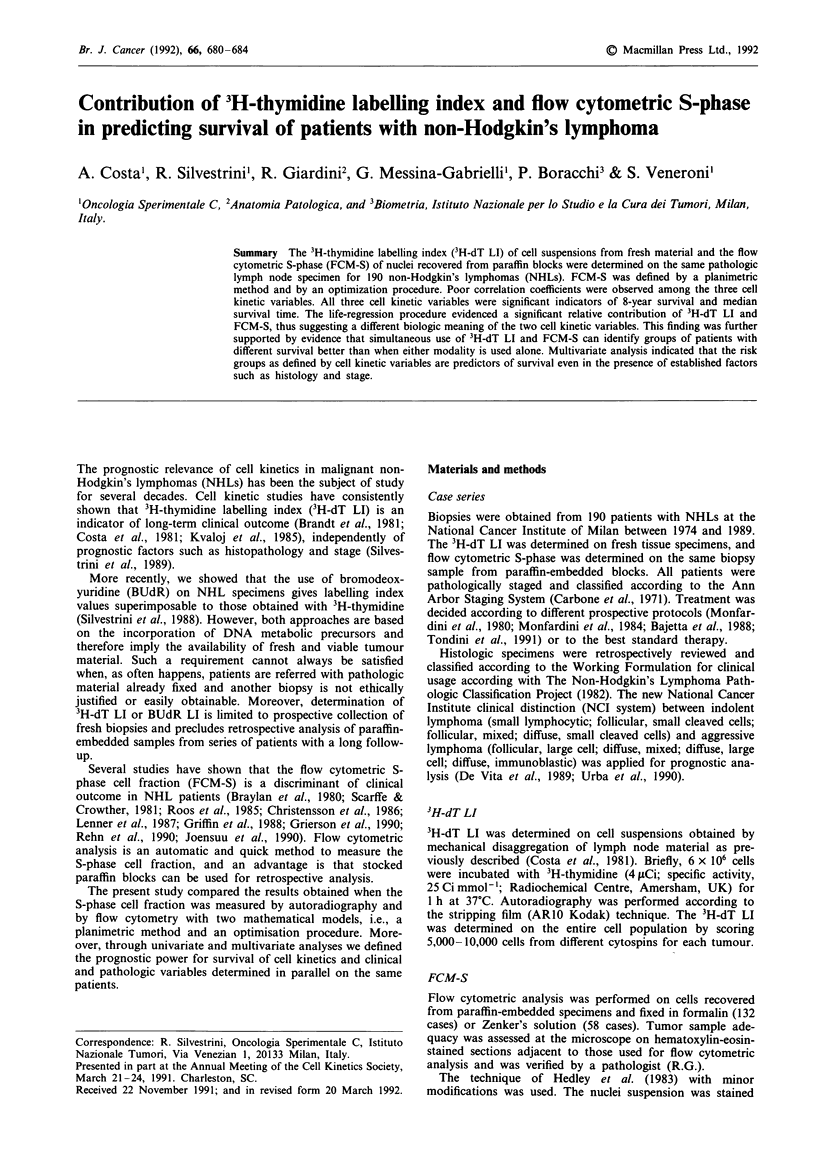

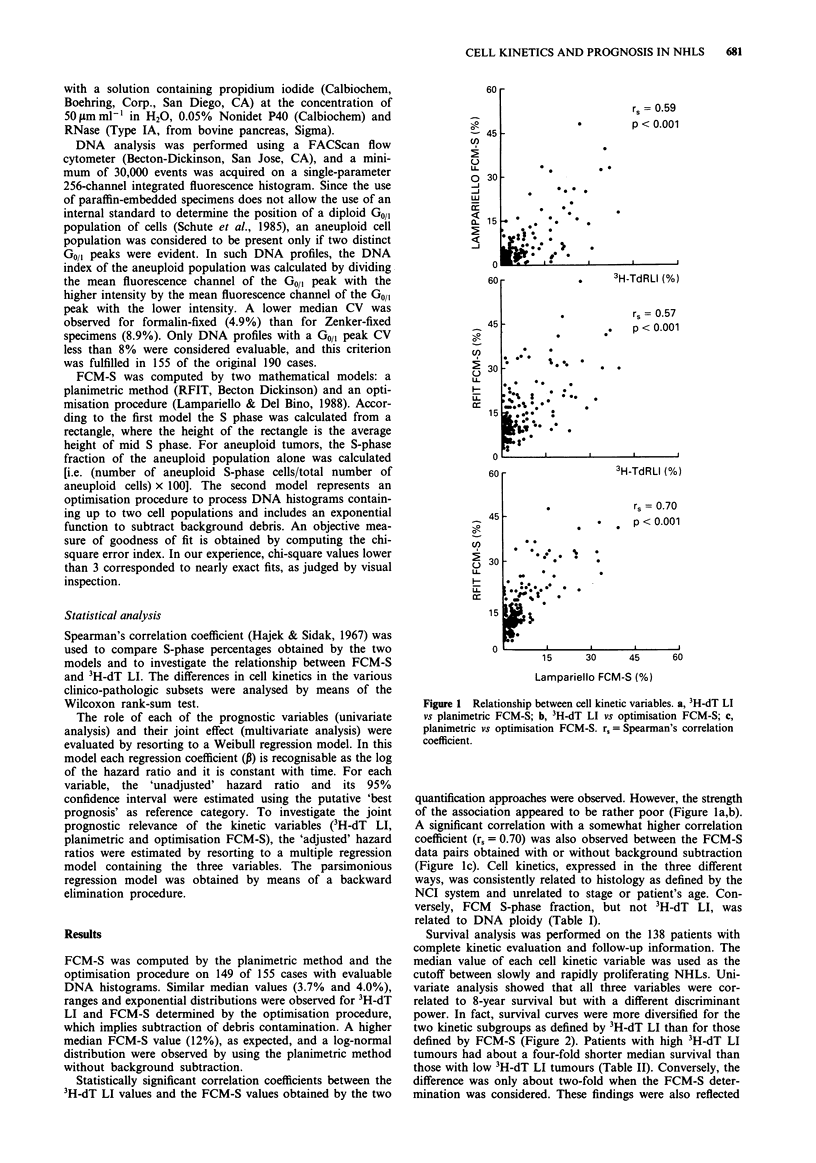

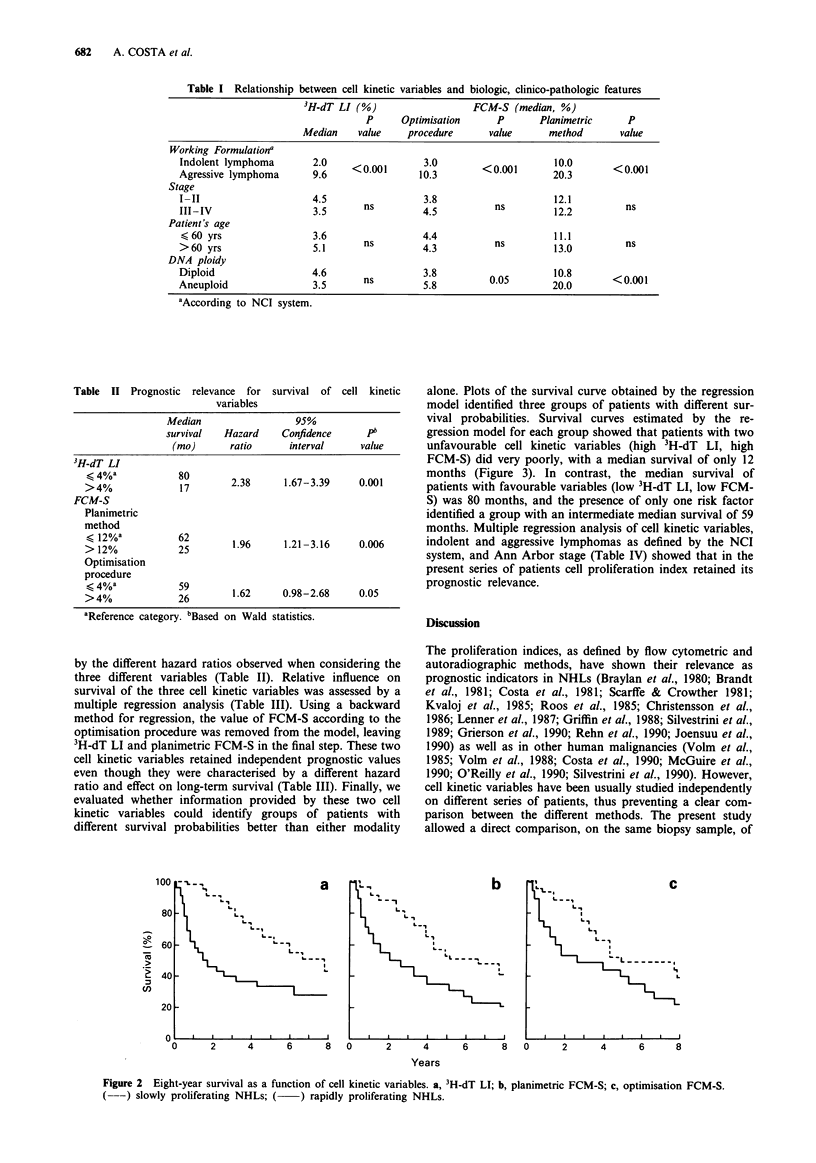

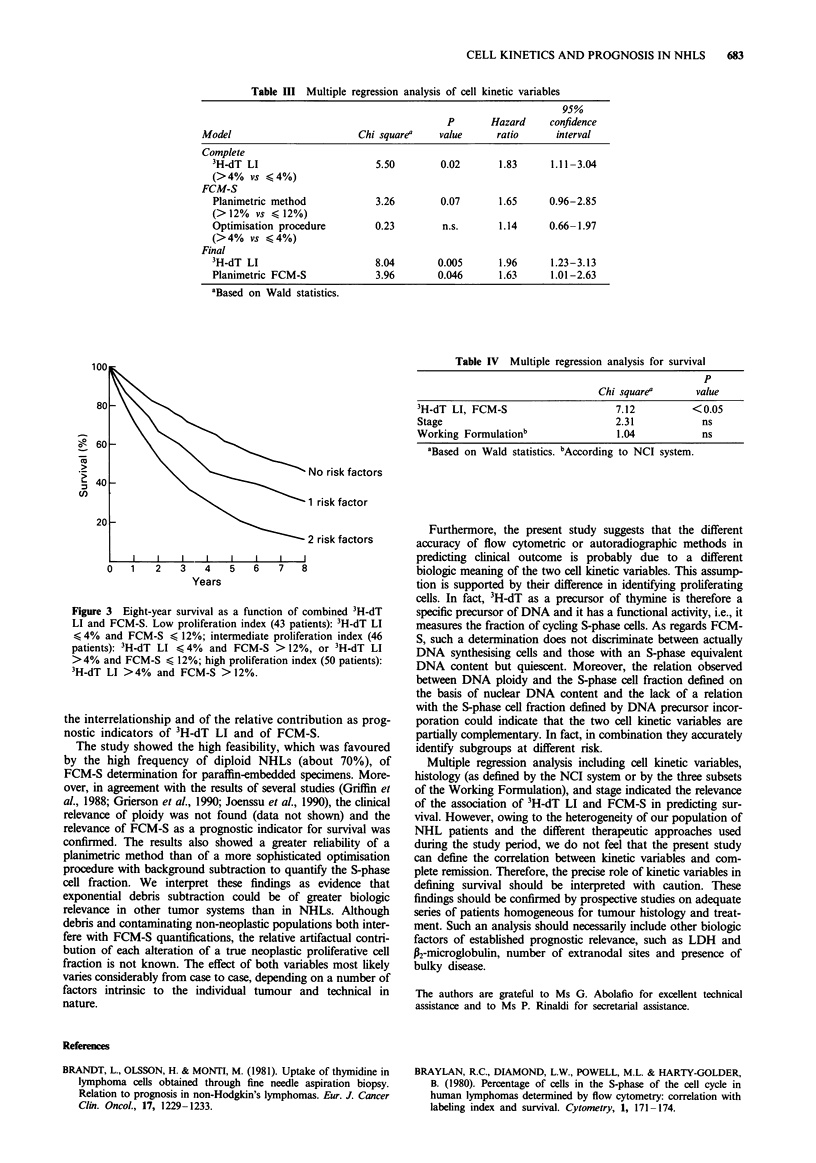

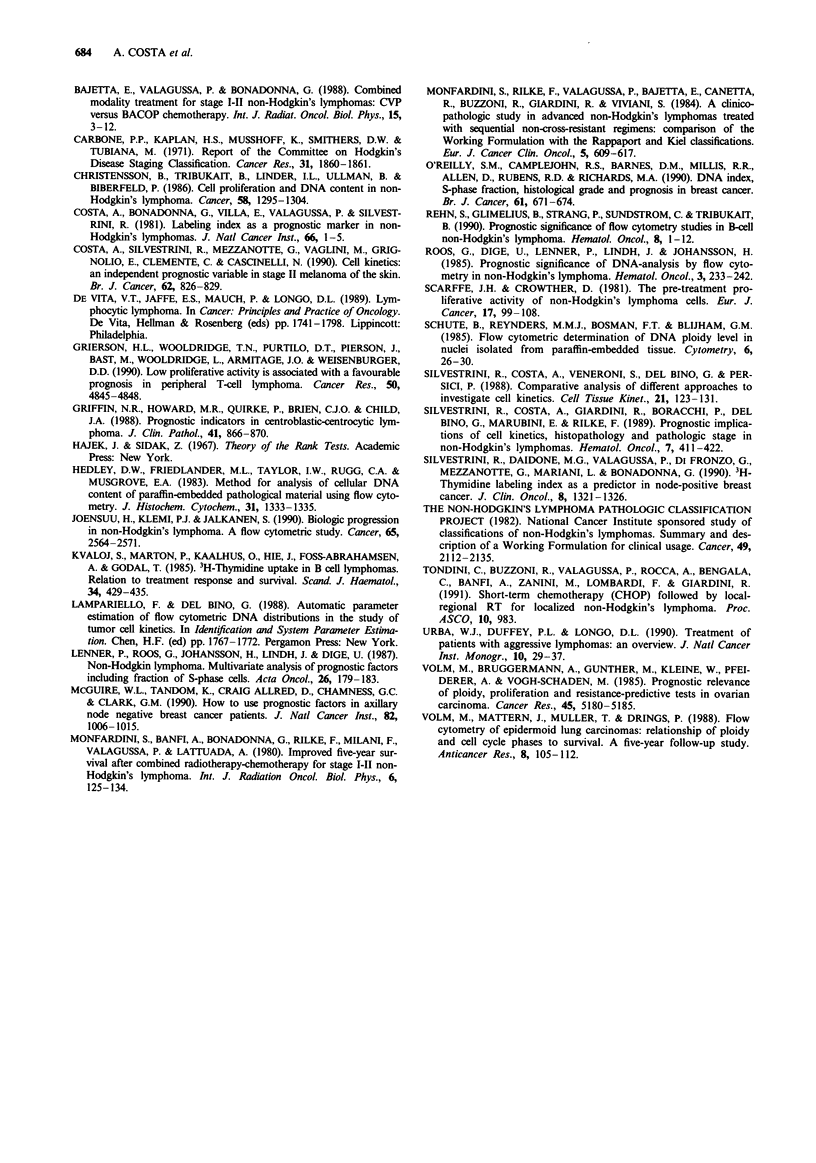


## References

[OCR_00593] Bajetta E., Valagussa P., Bonadonna G., Lattuada A., Buzzoni R., Rilke F., Banfi A. (1988). Combined modality treatment for stage I-II non-Hodgkin's lymphomas: CVP versus BACOP chemotherapy.. Int J Radiat Oncol Biol Phys.

[OCR_00579] Brandt L., Olsson H., Monti M. (1981). Uptake of thymidine in lymphoma cells obtained through fine-needle aspiration biopsy. Relation to prognosis in non-Hodgkin's lymphomas.. Eur J Cancer Clin Oncol.

[OCR_00585] Braylan R. C., Diamond L. W., Powell M. L., Harty-Golder B. (1980). Percentage of cells in the S phase of the cell cycle in human lymphoma determined by flow cytometry.. Cytometry.

[OCR_00599] Carbone P. P., Kaplan H. S., Musshoff K., Smithers D. W., Tubiana M. (1971). Report of the Committee on Hodgkin's Disease Staging Classification.. Cancer Res.

[OCR_00604] Christensson B., Tribukait B., Linder I. L., Ullman B., Biberfeld P. (1986). Cell proliferation and DNA content in non-Hodgkin's lymphoma. Flow cytometry in relation to lymphoma classification.. Cancer.

[OCR_00611] Costa A., Bonadonna G., Villa E., Valagussa P., Silvestrini R. (1981). Labeling index as a prognostic marker in non-Hodgkin's lymphomas.. J Natl Cancer Inst.

[OCR_00616] Costa A., Silvestrini R., Mezzanotte G., Vaglini M., Grignolio E., Clemente C., Cascinelli N. (1990). Cell kinetics: an independent prognostic variable in stage II melanoma of the skin.. Br J Cancer.

[OCR_00626] Grierson H. L., Wooldridge T. N., Purtilo D. T., Pierson J., Bast M., Wooldridge L., Armitage J. O., Weisenburger D. D. (1990). Low proliferative activity is associated with a favorable prognosis in peripheral T-cell lymphoma.. Cancer Res.

[OCR_00633] Griffin N. R., Howard M. R., Quirke P., O'Brien C. J., Child J. A., Bird C. C. (1988). Prognostic indicators in centroblastic-centrocytic lymphoma.. J Clin Pathol.

[OCR_00642] Hedley D. W., Friedlander M. L., Taylor I. W., Rugg C. A., Musgrove E. A. (1983). Method for analysis of cellular DNA content of paraffin-embedded pathological material using flow cytometry.. J Histochem Cytochem.

[OCR_00648] Joensuu H., Klemi P. J., Jalkanen S. (1990). Biologic progression in non-Hodgkin's lymphoma. A flow cytometric study.. Cancer.

[OCR_00653] Kvaløy S., Marton P. F., Kaalhus O., Høie J., Foss-Abrahamsen A., Godal T. (1985). 3H-thymidine uptake in B cell lymphomas--relationship to treatment response and survival.. Scand J Haematol.

[OCR_00664] Lenner P., Roos G., Johansson H., Lindh J., Dige U. (1987). Non-Hodgkin lymphoma. Multivariate analysis of prognostic factors including fraction of S-phase cells.. Acta Oncol.

[OCR_00669] McGuire W. L., Tandon A. K., Allred D. C., Chamness G. C., Clark G. M. (1990). How to use prognostic factors in axillary node-negative breast cancer patients.. J Natl Cancer Inst.

[OCR_00675] Monfardini S., Banfi A., Bonadonna G., Rilke F., Milani F., Valagussa P., Lattuada A. (1980). Improved five year survival after combined radiotherapy-chemotherapy for stage I-II non-Hodgkin's lymphoma.. Int J Radiat Oncol Biol Phys.

[OCR_00682] Monfardini S., Rilke F., Valagussa P., Bajetta E., Canetta R., Buzzoni R., Giardini R., Viviani S. (1984). A clinicopathologic study in advanced non-Hodgkin's lymphomas treated with sequential non-cross-resistant regimens: comparison of the working formulation with the Rappaport and Kiel classifications.. Eur J Cancer Clin Oncol.

[OCR_00690] O'Reilly S. M., Camplejohn R. S., Barnes D. M., Millis R. R., Allen D., Rubens R. D., Richards M. A. (1990). DNA index, S-phase fraction, histological grade and prognosis in breast cancer.. Br J Cancer.

[OCR_00696] Rehn S., Glimelius B., Strang P., Sundström C., Tribukait B. (1990). Prognostic significance of flow cytometry studies in B-cell non-Hodgkin lymphoma.. Hematol Oncol.

[OCR_00701] Roos G., Dige U., Lenner P., Lindh J., Johansson H. (1985). Prognostic significance of DNA-analysis by flow cytometry in non-Hodgkin's lymphoma.. Hematol Oncol.

[OCR_00705] Scarffe J. H., Crowther D. (1981). The pre-treatment proliferative activity of non-Hodgkin's lymphoma cells.. Eur J Cancer.

[OCR_00710] Schutte B., Reynders M. M., Bosman F. T., Blijham G. H. (1985). Flow cytometric determination of DNA ploidy level in nuclei isolated from paraffin-embedded tissue.. Cytometry.

[OCR_00721] Silvestrini R., Costa A., Giardini R., Boracchi P., Del Bino G., Marubini E., Rilke F. (1989). Prognostic implications of cell kinetics, histopathology and pathologic stage in non-Hodgkin's lymphomas.. Hematol Oncol.

[OCR_00718] Silvestrini R., Costa A., Veneroni S., Del Bino G., Persici P. (1988). Comparative analysis of different approaches to investigate cell kinetics.. Cell Tissue Kinet.

[OCR_00727] Silvestrini R., Daidone M. G., Valagussa P., Di Fronzo G., Mezzanotte G., Mariani L., Bonadonna G. (1990). 3H-thymidine-labeling index as a prognostic indicator in node-positive breast cancer.. J Clin Oncol.

[OCR_00747] Urba W. J., Duffey P. L., Longo D. L. (1990). Treatment of patients with aggressive lymphomas: an overview.. J Natl Cancer Inst Monogr.

[OCR_00754] Volm M., Brüggemann A., Günther M., Kleine W., Pfleiderer A., Vogt-Schaden M. (1985). Prognostic relevance of ploidy, proliferation, and resistance-predictive tests in ovarian carcinoma.. Cancer Res.

[OCR_00758] Volm M., Mattern J., Müller T., Drings P. (1988). Flow cytometry of epidermoid lung carcinomas: relationship of ploidy and cell cycle phases to survival. A five-year follow up study.. Anticancer Res.

